# Hepatitis B and celiac disease

**Published:** 2010-12-01

**Authors:** Hugh James Freeman

**Affiliations:** 1 University of British Columbia, Vancouver, BC, Canada

Dear Editor,

Evidence suggests a linkage between different environmental agents and the development of celiac disease-an immune-mediated disorder-in those with a specific genetic predisposition (HLA-DQ2, HLA-DQ8) who exposed to gluten-a major storage protein in wheat and other grains. Infections, particularly viral agents, have been hypothesized to induce or exacerbate immune-mediated disorders, possibly through a mechanism of molecular mimicry [1][2]. Others have suggested that treatment of chronic viral infections (i.e., with α-interferon) or vaccines administered for prevention may be further triggers the process [1]. In celiac disease, a host of chronic immunologically-based liver disorders may occur [3]. Some early studies noted a relatively high frequency of hypertransaminasemia in celiac disease that usually resolved with a gluten-free diet [4]. Failure of raised transaminase values to fall with a gluten-free diet led to eventual detection of chronic infection with hepatitis B or C viruses leading to the hypothesis that infection with hepatitis B or C viruses may trigger or "unmask" the underlying celiac disease.In the study conducted by Leonardi and Rosa [5], serological testing for celiac disease antibodies was performed in 60 patients with chronic hepatitis B carrier state or a prior, but recovered, hepatitis B infection. Some of the carriers were treated for 12 months with α-interferon. Selective IgA deficiency was not present. In all 60 patients, IgA endomysial antibodies (EMA)-using a monkey esophagus immunofluorescence assay-and IgA tissue transglutaminase antibodies (tTGA)-using a commercial ELISA assay-were negative.In summary, a linkage between hepatitis B infection and serologically-defined celiac disease could not be established. Moreover, there was no evidence that α-interferon could activate celiac disease. Although the investigators acknowledge the limited sample size of their hepatitis B cohort, the study still provides intriguing data. Future studies might also need to directly examine intestinal biopsies, not only for histological evidence of celiac disease, but for evidence of prior viral "footprints" [6], including identification of specific viral sequences.

## References

[1] Molina V, Shoenfeld Y (2005). Infection, vaccines and other environmental triggers of autoimmunity. Autoimmunity.

[2] Plot L, Amital H (2009). Infectious associations of Celiac disease. Autoimmun Rev.

[3] Freeman H (2010). Hepatic manifestations of celiac disease. Clinical and Experimental Gastroenterology.

[4] Bardella MT, Fraquelli M, Quatrini M, Molteni N, Bianchi P, Conte D (1995). Prevalence of hypertransaminasemia in adult celiac patients and effect of gluten-free diet. Hepatology.

[5] Leonardi S, La Rosa M (2010). Are Hepatitis B Virus and Celiac Disease Linked?. Hepat Mon.

[6] Schattner A, Rager-Zisman B (1990). Virus-induced autoimmunity. Reviews of infectious diseases.

